# Detection of environmental pollutant cadmium in water using a visual bacterial biosensor

**DOI:** 10.1038/s41598-022-11051-9

**Published:** 2022-04-27

**Authors:** Chang-ye Hui, Yan Guo, Han Li, Chao-xian Gao, Juan Yi

**Affiliations:** 1 Department of Pathology and Toxicology, Shenzhen Prevention and Treatment Center for Occupational Diseases, Shenzhen, 518020 China; 2 National Key Clinical Specialty of Occupational Diseases, Shenzhen Prevention and Treatment Center for Occupational Diseases, Shenzhen, 518020 China; 3 College of Lab Medicine, Hebei North University, Zhangjiakou, 075000 China

**Keywords:** Microbiology techniques, Environmental biotechnology

## Abstract

Cadmium (Cd) contamination in water and soil is considered an environmental pollutant. Food crops can absorb and accumulate bioavailable Cd. Continuous monitoring of Cd levels in the environment can minimize exposure and harm to humans. Visual pigments have been demonstrated to have great potential in the development of minimal-equipment biosensors. In the present study, a metabolically engineered bacterium was employed to produce blue-purple pigment violacein responsive to toxic Cd(II). The high stability of the bisindole pigment contributed to determining the violacein at wavelengths of 578 nm. Visual and quantifiable signals could be captured after a 1.5-h Cd(II) exposure. This novel biosensor showed significantly stronger responses to Cd(II) than to other heavy metals including Pb(II), Zn(II), and Hg(II). A significant increase in pigment signal was found to respond to as low as 0.049 μM Cd(II). The naked eye can detect the color change when violacein-based biosensor is exposed to 25 μM Cd(II). A high-throughput method for rapid determination of soluble Cd(II) in environmental water was developed using a colorimetric microplate.

## Introduction

Environmental pollution arising from Cd is a worldwide concern because of its persistence, bioaccumulation, and highly toxic properties^1^. Cd and its various compounds, as settled, soluble, bio-adsorbed, or bio-accumulated forms, are dispersed in the ecosystem, so the determination of bioavailable Cd is useful in health risk assessment of Cd pollution^2^. As an effective supplement to instrumental methods, the whole-cell biosensors have been well demonstrated to be powerful tools for predicting accumulation, translocation, and ecotoxicological effects of heavy metal pollution^3^.

So far, bioluminescent, chemiluminescent, fluorescent, and traditional colorimetric detection systems have been successfully employed in developing bacterial whole-cell biosensors toward heavy metals including Cd(II)^4–9^. Pigmented metabolites are visible to the naked eye, and so the use of colored pigments as biosensing readouts holds great promise in the design of heavy metal biosensors^10–13^. The visible signal output is able to be interpreted without the need for complicated instruments, enabling them as powerful tools especially useful in low-resource areas.

Violacein, as a hydrophobic chromogenic pigment produced by environmental bacteria, displays a charming purple-blue hue^14^. The violacein biosynthetic pathway has been extensively clarified and employed in several recent studies^15–17^. Whole-cell biosensors using violacein as a visible readout have been validated in detecting essential micronutrients such as zinc in biological samples^18^, as well as toxic lead and toxic mercury in environmental samples^19,20^. In this study, violacein production is performed by the heterologous biosynthetic genes expressed in bacterial biosensor under the control of Cd(II) sensory elements. The novel violacein-based biosensor could efficiently respond to low levels and a wide concentration range of Cd(II). Furthermore, a high-throughput 96-well microplate bioassay was established and validated in monitoring bioaccessible Cd(II) in environmental water samples in a visible and stable manner.

## Materials and method

### Bacterial strain, vectors, and agents

*Escherichia coli* (*E. coli*) TOP10 was used as the bacterial host. Engineered bacteria were cultured in Luria–Bertani (LB) broth (10 g/L tryptone, 5 g/L yeast extract, and 10 g/L NaCl) supplemented with 50 μg/mL ampicillin. Stock solutions of CdCl_2_, Pb(NO_3_)_2_, ZnSO_4_, and HgCl_2_ were freshly prepared using analytical grade reagents and purified water.

### Assembly of the biosensing construct

The plasmid pET-vio with a violacein biosynthetic gene cluster (*vioABCDE*) inserted into the *Nde*I/*Sac*I sites of pET-21a was previously constructed^20^. The plasmid pCadR^21^ was digested with *Bgl*II and *Xba*I, and the resultant fragment containing the Cd(II) sensory module, the *cadR* gene and its divergent *cad* promoter, was inserted into the same sites of pET-vio to generate pPcad-vio. The resultant biosensing construct pPcad-vio was used for transformation into *E. coli* TOP10 and spread on LB agar plates containing 50 μg/mL ampicillin. After inverted incubation at 37 °C for 12 h, single colonies were picked up for the following studies. *E. coli* TOP10 harboring the biosensing construct pPcad-vio is expected to sense bioavailable Cd(II) using biosynthetic violacein as a visual output signal.

### Stability investigation of violacein-derived signal

The overnight LB culture of TOP10/pPcad-vio was used to inoculate fresh LB medium (1% v/v inoculum). After 3 h incubation at 37 °C with shaking at 250 rpm, engineered TOP10/pPcad-vio in early exponential phase (OD_600_ = 0.17) was induced with 25 μM Cd(II) for 3 h, and the intracellular violacein agglomerates were extracted with butanol as described previously^19^. The butanol extraction phase was placed at 37 °C and sampled at regular time intervals. The visible light absorption spectrum of the sample was measured in a microplate reader (BioTek Epoch, USA). A 300–750 nm scanning wavelength range with an interval of 2 nm was set.

### Analysis of response pattern of whole-cell biosensor

To evaluate the time-dependent response of biosensor toward different concentrations of Cd(II), overnight culture of TOP10/pPcad-vio was inoculated using 1% v/v inoculum to 3 mL of LB medium and grown at 37 °C for 3 h. A final concentration of 0, 0.15, and 1.5 μM Cd(II) was supplemented into the early exponential-phase culture, followed by a 6-h incubation and sampled at regular time intervals. One milliliter culture was mixed with 400 μL butanol and vortexed violently for 2 min. The upper extract was prepared by centrifugation at 8000*g* for 2 min. To determinate the content of violacein in the butanol extraction phase, aliquots of 100 μL were transferred into a 96-well microplate and measured at 578 nm using a microplate reader.

### Detection selectivity assay

To evaluate the detection selectivity of the biosensor, stock solutions of Cd(II), Pb(II), Zn(II), and Hg(II) were added into the early exponential-phase culture of TOP10/pPcad-vio at a final concentration of 0, 1.25, 2.5, 5, 10, and 20 μM. This was then followed by a 1.5-h culture at 37 °C before bacterial density and pigment signal were determined. Aliquots of culture containing 100 μL were pipetted into a microplate directly and measured at 600 nm for bacterial density. After butanol extraction, the violacein-derived signal was determined as described above.

### Cd(II) detection with the developed whole-cell biosensor

To investigate the performance of the biosensor toward increased concentrations of Cd(II), the early exponential-phase cultures of TOP10/pPcad-vio were exposed to 0, 0.0061, 0.012, 0.024, 0.049, 0.098, 0.195, 0.39, 0.78, 1.56, 3.125, 6.25, 12.5, 25, 50, 100, 200, 400, and 800 μM Cd(II) at 37 °C using a double dilution method as described previously^21^. After a 1.5-h induction, aliquots of 100 μL culture were read at 600 nm to determine the bacterial density, and aliquots of 100 μL butanol extract were read at 578 nm to determine the violacein-derived signal.

### Detection of environmental water samples

To validate the capability of the biosensor to detect bioavailable Cd(II) in environmental water samples, engineered TOP10/pPcad-vio was cultured in LB medium prepared with purified water, tap water, and two kinds of surface water as described previously^11^. The early exponential-phase culture were spiked with 0, 0.39, 0.78, 1.56, 3.125, 6.25, and 12.5 μM Cd(II) using a double dilution method. Bacterial density and pigment signal were measured after 1.5 h of incubation at 37 °C.

## Results and discussion

### Assembly of violacein-based Cd(II) biosensor

**Figure 1:**
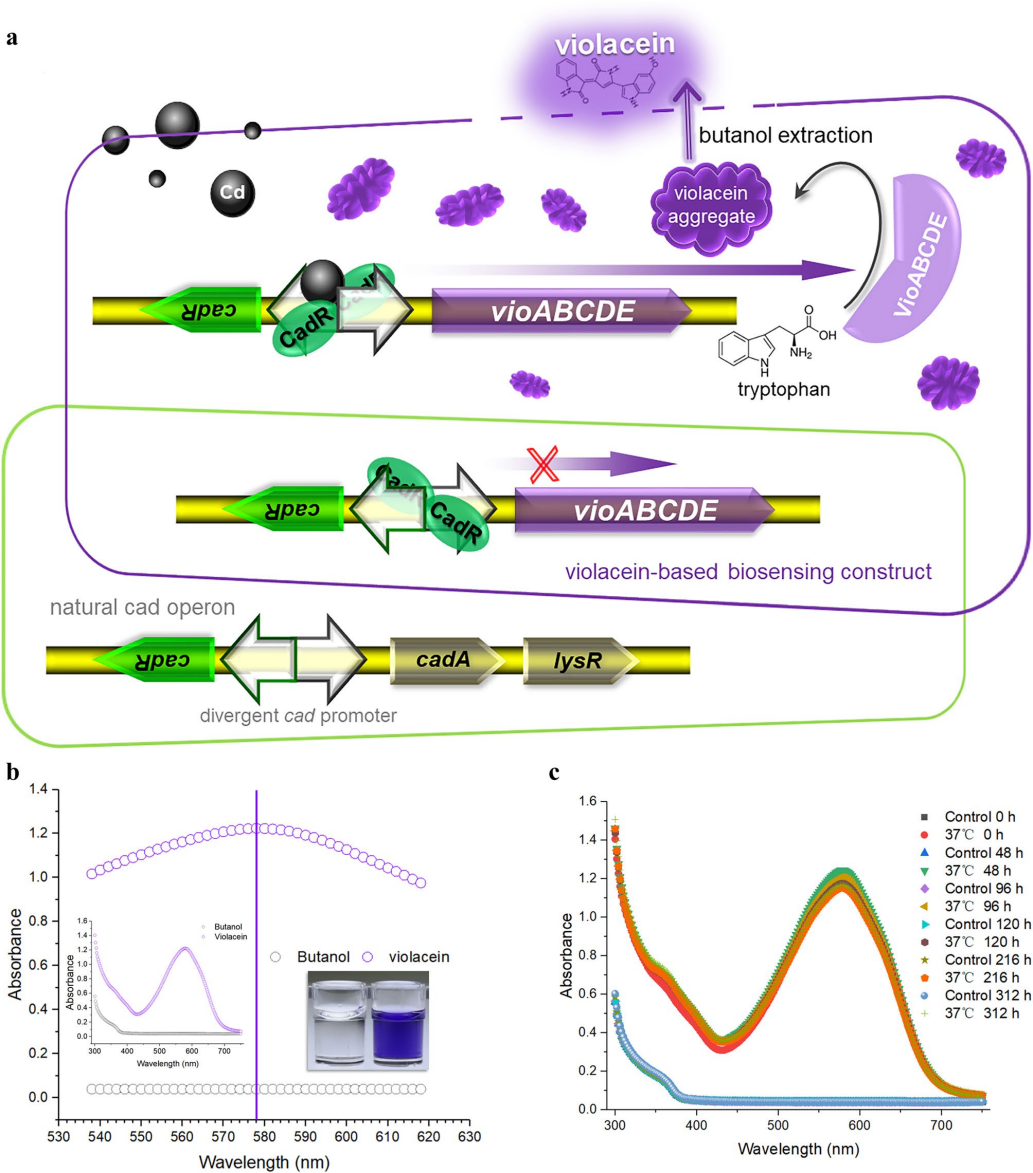
**(a)** The violacein biosynthesis triggered by Cd(II) sensory element. (**b)** The maximum absorption wavelength of violacein produced by Cd(II)-induced TOP10/pPcad-vio. The left inset is the whole visible absorption spectrum ranging from 300 to 750 nm, and the right inset is the butanol-soluble violacein. (**c)** The stability of violacein-based pigment signal. The butanol-soluble violacein was placed at 37 °C and sampled at regular time intervals for the spectrum scanning. Experiments were performed four times with similar results and one of the representative experimental results is shown.

As a secondary metabolite, the bisindole violacein is generated by the condensation of two L-tryptophan molecules, and this reaction was usually catalyzed by five biosynthetic enzymes in diverse bacteria^22^. The violacein biosynthesis pathway has been heterogeneously reconstructed using the *vioABCDE* gene cluster derived from *Chromobacterium violaceum* in *E. coli*^20^. The metalloregulator CadR originally characterized in *Pseudomonas putida*^23^, has been successfully employed as the sensory element to develop Cd(II)-responsive biosensors with fluorescent proteins as the output signals in our previous studies^5,21,24^. In the present study, a Cd(II) biosensing construct was assembled by employing the CadR and its divergent promoter as the Cd(II) sensory module and the *vioABCDE* gene cluster as the visual reporter module. As shown in Fig. 1a, the expression of pigment biosynthetic genes is activated when the whole-cell biosensor is exposed to bioavailable Cd(II). Hydrophobic violacein cannot be secreted by the host and accumulates in the cell. Violacein can be released after butanol treatment and then quantified using a colorimetric method. Violacein is vibrant blue-purple and has maximum absorbance at about 578 nm (Fig. 1b). Furthermore, violacein in the butanol was demonstrated to be very stable. The visible adsorption spectrum of pigment remains almost unchanged over a 13-day period (Fig. 1c). Given the light absorption characteristics and stability of violacein described above, the pigment content in the butanol was determined at 578 nm in the following study.

### Response pattern of violacein-based biosensor toward Cd(II)

**Figure 2:**
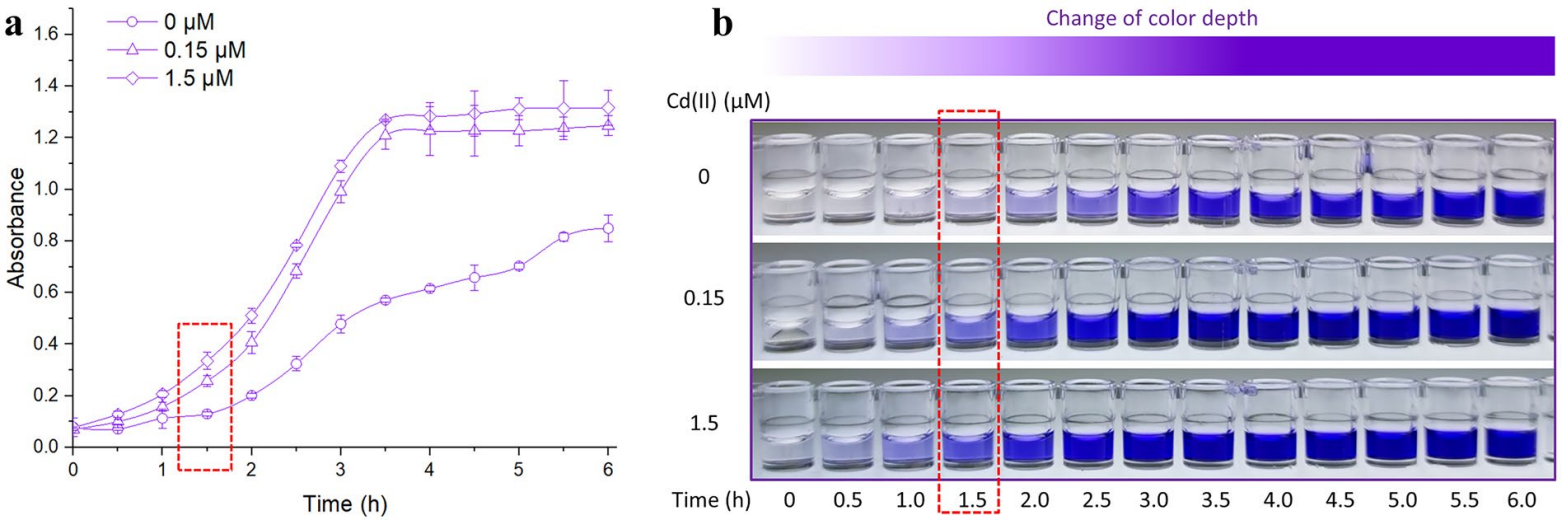
**(a)** Time-response curves of the biosensor upon exposure to increased concentrations of Cd(II). The results are shown as the mean of four independent assays ± SD. (**b)** The butanol extraction phases containing violacein were prepared at regular time intervals before read at 578 nm in a microplate reader. The representative image from four independent experiments is shown here. The red dotted box shows the pigment signal derived from the biosensor after a 1.5-h Cd(II) induction.

The basal expression has been shown to be common in most identified heavy metal resistance operons and this property was believed to facilitate bacterial survival in the unpredictable and complex environment^25,26^. High-background biosensing platforms for cadmium have been reported due to the leaky expression of fluorescent reporters in these artificial cadmium resistance operons^5,7,21^. Pigment reporter, which is produced by biosynthetic enzymes catalysis, can be expected to constantly accumulate along with prolonging induction^15^. This property has been proved to improve the sensitivity of pigment-based biosensor^13^. However, the background expression signal may also be amplified^20^. The target optimal induction time is a strong biosensing signal with the lowest background signal. As shown in Fig. 2a, both of the Cd(II)-induced violacein signals increased with the extension of induction time, and reached their maximum strengths after a 3.5-h induction. The leaky production of violacein was not obvious after 1.5-h incubation, however, the accumulation of violacein constantly increased since 2 h induction. The color change of butanol phase could be distinguished easily by the naked eye (Fig. 2b). Considering a low background and a strong enough Cd(II)-induced visual signal, a 1.5-h induction was chosen in the following study.

### Response selectivity of violacein-based biosensor

**Figure 3:**
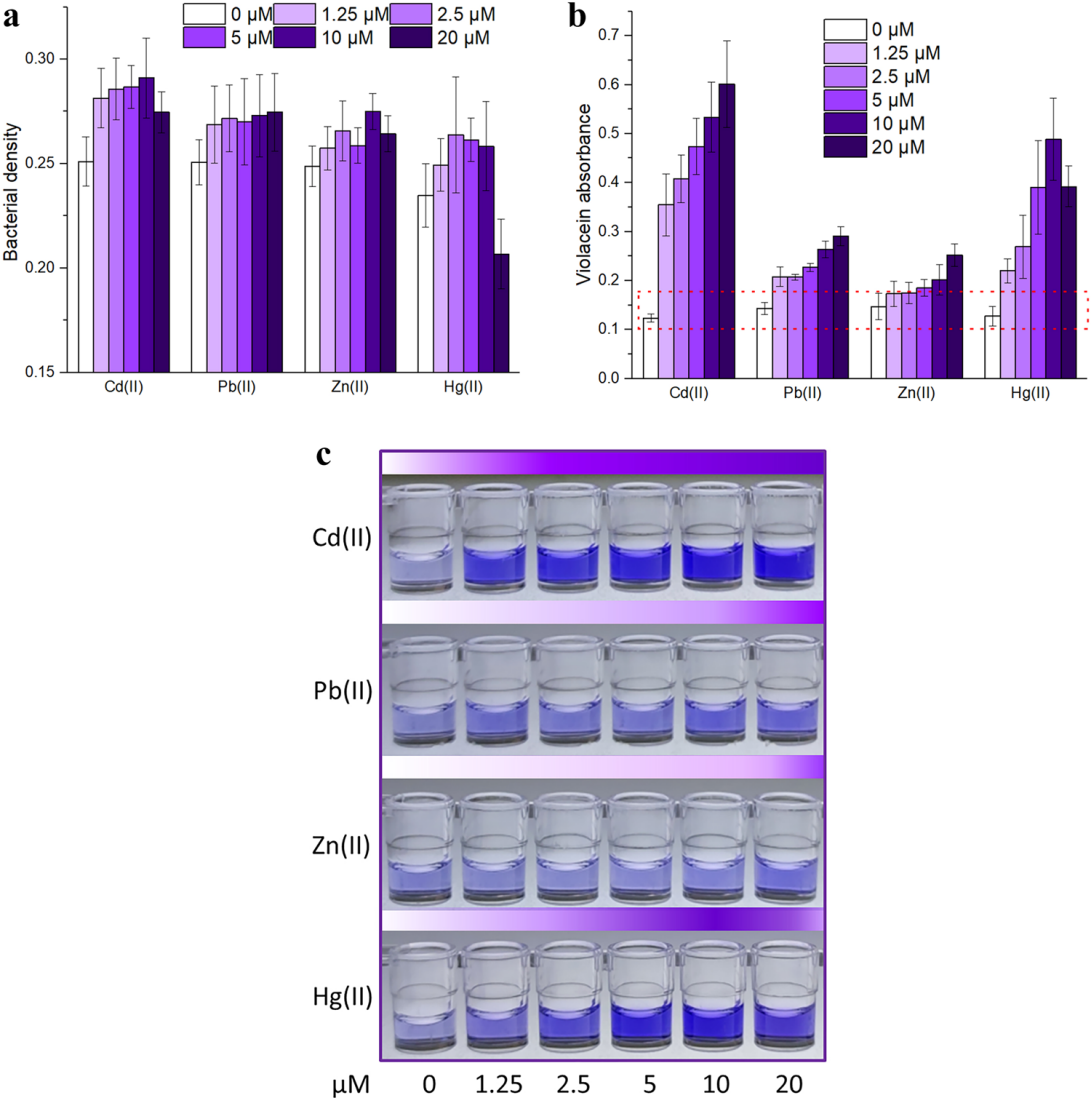
The performance of biosensors toward different metal ions. TOP10/pPcad-vio during the early exponential phase was exposed to four kinds of metal ions. After being cultured at 37 °C for 1.5 h, the bacterial densities (**a**) and the violacein signals (**b**) were measured at 600 nm and 578 nm, respectively. The red dotted box shows the pigment signals derived from the biosensor without exposure to metal ions. (**c)** A representative photo of butanol extraction phases from four independent experiments with similar results is shown here.

For the investigation of biosensing selectivity, *E. coli* TOP10 carrying pPcad-vio was exposed to 0, 1.25, 2.5, 5, 10, and 20 μM Cd(II), Pb(II), Zn(II), or Hg(II). Violacein aggregates accumulated in bacterial cells could exert a certain influence on the determination of bacterial density at 600 nm^20^. For this reason, bacterial densities were slightly increased in all groups with violacein overproduction (Fig. 3a). Bacterial biosensor was responsive to Cd(II) > Hg(II), almost non-responsive to Pb(II) and Zn(II) (Fig. 3b). Importantly, the biosensor strongly responded to Cd(II) in a dose–response relationship ranging from 0 to 20 μM. Furthermore, the biosensor also responded to Hg(II) in a dose–response relationship ranging from 0 to 10 μM. Owning to the poor selectivity of metalloregulator CadR, non-specific response toward Hg(II) was also found in previously developed CadR-based biosensors^4,5,8,27^. The violacein-based reporter was demonstrated to not interfere with the metal specificity of metalloregulator CadR. A color deepening in butanol phases is obvious in Cd(II) and Hg(II) exposure groups (Fig. 3c). The decrease in bacterial density and violacein absorbance upon exposure to 20 μM Hg(II) was possibly attributed to significant cytotoxicity^28^.

### Response of violacein-based biosensor toward Cd(II) with systematically varied concentrations

**Figure 4:**
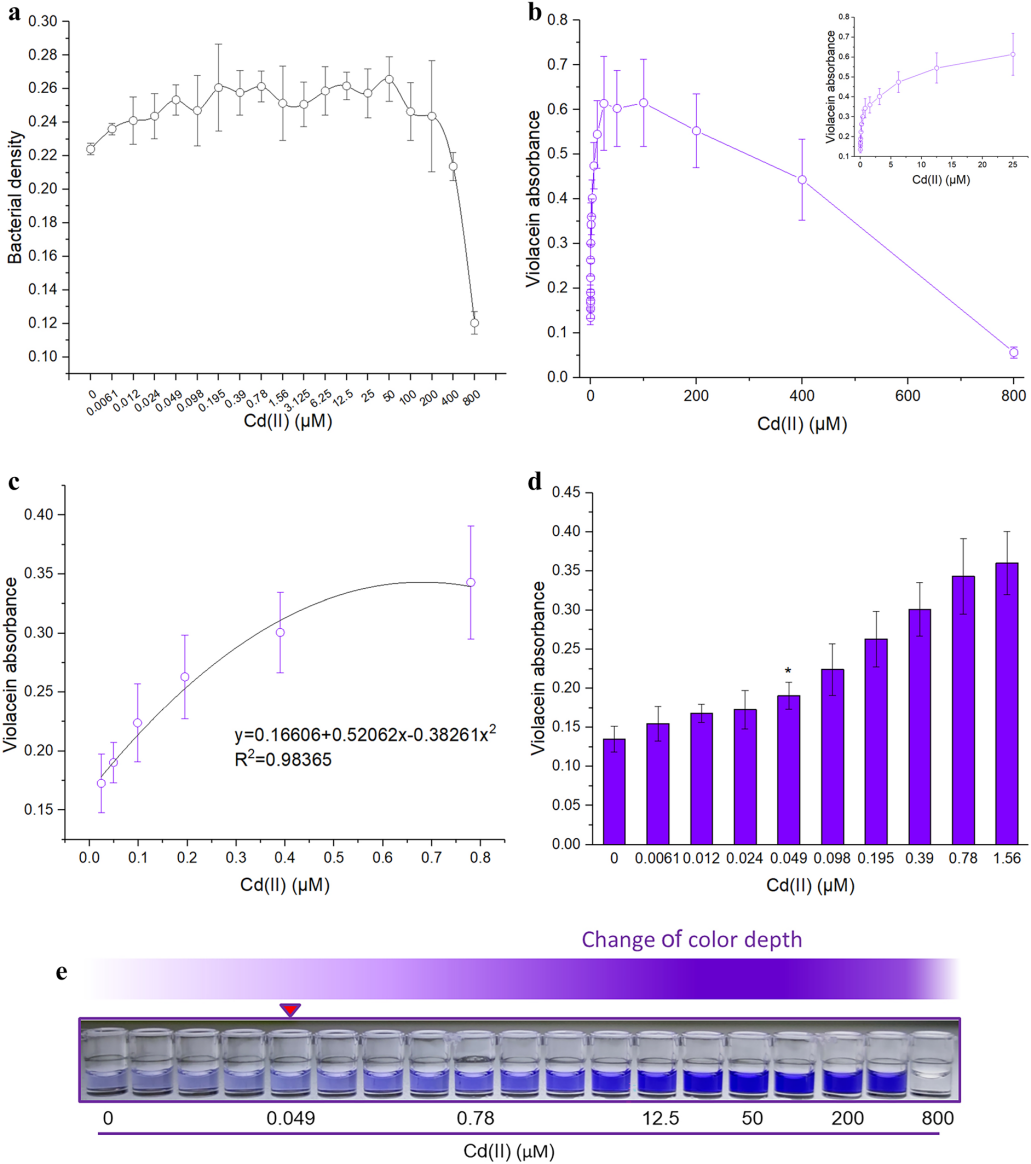
The performance of biosensor exposed to increased concentrations of Cd(II). (**a)** TOP10/pPcad-vio in early exponential phase from 0 to 800 μM Cd(II). After being cultured at 37 °C for 1.5 h, bacterial cell densities were measured at 600 nm. (**b)** The responses of biosensor toward 0–800 μM Cd(II). The inset shows the dose–response curve within 0–25 μM Cd(II). (**c)** Regression analysis of the relationship between violacein-derived signal and Cd(II) concentration (0.024–0.78 μM). (**d)** The detection sensitivity of the biosensor. The asterisk shows the limit of detection, which was defined as the lowest metal ion concentration that induced a significantly enhanced production of violacein (background + 3 × SD). (**e)** A representative photo from four independent experiments with similar results is shown here.

To characterize the overall response of the biosensor toward a wide concentration range of Cd(II), *E. coli* TOP10 harboring pPcad-vio was exposed to 0–800 μM Cd(II) using a double dilution method. Consistent with previous studies^21^, the cytotoxicity of Cd(II) was high upon exposure to above 200 μM Cd(II). Bacterial density decreased dramatically when the cytotoxicity of high concentrations of Cd(II) was obvious (Fig. 4a). The biosensing response was found to increase initially (0–25 μM), to maintain the maximum stable response (25–100 μM), and then to decrease steadily (100–800 μM) (Fig. 4b). Such an overall response of bacterial biosensor was believed to be determined by the molecular response properties of metalloregulators^25^. In the first stage, the biosensing signal constantly increases with the increase of the concentration of low-level intracellular cognate metal. In the second stage, the biosensing signal maintains stable when the metal binding sites of metalloregulators are saturated. In the third stage, the biosensing signal will decrease sharply when cytotoxicity of high-level cognate metal is obvious. Both linear and non-linear regression analyses were previously proposed to quantify the content of cognate metals using whole-cell biosensors^13,20^. Although the absorbance of violacein was proportional to the Cd(II) concentration ranging from 0 to 25 μM, a good non-linear response (R^2^ = 0.98365) to Cd(II) was only found in a low, narrow concentration range (Fig. 4c). Although this response property limits the precise quantitative assessment of bioavailable Cd(II), the biosensor has a great potential in approximately quantitating toxic Cd(II).

The detection limit could be defined as the lowest metal concentration that evokes a significant increase in biosensing signal (background + 3 × SD)^7,11^. The detection limit using bacterial biosensor in the present investigation was about 0.049 μM (Fig. 4d), which is lower than early reported CadR-based biosensor employing fluorescent reporters^5,21^, but still higher than the criteria maximum concentration (CMC) of cadmium in freshwater (about 0.016 μM) recommended by the United States Environmental Protection Agency (USEPA)^2^. However, the detection limit of violacein-based biosensors is similar to the permissible limits for cadmium in drinking water recommended by China, European Communities (EC), and World Health Organization (WHO)^2^.

Previously developed bacterial whole-cell biosensors toward Cd(II) were summarized in Table 1. Although the biosensors using fluorescence protein reporters usually showed high detection limits, various genetic circuits (amplification, logic gate, toggle switch) have been demonstrated to facilitate the improved detection sensitivity^29,30^. Compared with fluorescence proteins, enzyme reporters including luciferase^31,32^ and β-galactosidase^33^ have been demonstrated to significantly enhance the detection sensitivity. However, the determination of two enzyme reporters is highly dependent on extra substrates, expensive instruments, and professional technicians. These disadvantages seriously limit their practical applications. Naked-eye recognition of pigments induced by heavy metals has been demonstrated to be an alternative to traditional reporters^2^. We recently developed an indigoidine-based biosensor which could detect Cd(II) as low as 0.024 μM^13^. The biosynthesis of water soluble indigoidine was dose-dependently induced by bioavailable Cd(II). Compared with the instable indigoidine-derived signal, violacein is highly stable, which is more convenient for detection.

**Table 1:** Comparison of developed whole-cell biosensors toward bioavailable Cd(II).

Host cells	Sensory element	Reporter	Detection range (μM)	LOD (μM)	Specificity	Ref
*Escherichia coli*	P*znt*A	Luciferase	0.01–0.3	0.01	Pb(II) and Hg(II)	^31^
	P*znt*A	LacZ	0.025–10	0.025	Cd(II), Zn(II), and Hg(II)	^33^
	P*znt*A	GFP	0.045–35.7	ND	Cd(II), Zn(II), and Hg(II)	^34^
	P*znt*A	eGFP	0.89–44.64	ND	Cd(II), Zn(II), and Pb(II)	^35^
		RFP	8.93–267.8	ND		
	Logic gated P*znt*A	GFP	12.3–333	ND	Cd(II) and Zn(II)	^36^
	P*znt*A	eGFP, RFP	8.93–89.29	ND	Cd(II), Zn(II), and Cr(III)	^37^
	P*cad*CA	GFP	0.09–0.45	0.09	Cd(II), Zn(II), and Pb(II)	^7^
	P*cad*CA	eGFP	3	3–30	Cd(II) and Pb(II)	^9^
	A polycistronic unit, P*cad*R	RFP, eGFP, and LacZ	0.1–1.56	0.1	Cd(II) and Hg(II)	^5^
	P*cad*CA, P*cad*R	RFP, eGFP	0.05–400	0.05	Cd(II), Pb(II) and Hg(II)	^21^
	A dual-sensing element, P*cad*R	RFP	0–200	0.1	Cd(II) and Hg(II)	^24^
	P*cad*R	Indigoidine	0–200	0.024	Cd(II) and Hg(II)	^13^
		RFP	0–200	0.78	Cd(II), Pb(II) and Hg(II)	
	P*cad*R	Violacein	0–25	0.049	Cd(II) and Hg(II)	This study
*Psuedomonas*	A toggle circuit, P*cad*R	GFP	0.01–1	0.01	ND	^29^
	P*cad*R	GFP	0.09–90	0.09	Cd(II), Pb(II) and Hg(II)	^8^
	A T7 RNAP circuit, P*cad*R	RFP	0.01–10	0.01	ND	^30^
*Staphylococcus aureus*	P*cad*CA	Luciferase	0.107–0.89	0.107	Cd(II), Pb(II), and Sb(III)	^32^
*Bacillus subtilis*			0.007–0.035	0.007		
*Shewanella oneidensis*	P*cad*R	GFP	0.09–90	0.09	Cd(II), Pb(II) and Hg(II)	^8^

*ND* not determined, *LOD* limit of detection, *lacZ* β-galactosidase, *GFP* green fluorescent protein, *eGFP* enhanced green fluorescent protein, *RFP* red fluorescent protein.

Although the results suggested that violacein-based Cd(II) biosensor was not suitable for accurate quantitative analysis, the blue-purple color in the butanol phase was significantly deepened with the increase of Cd(II) concentrations, especially at a metal concentration higher than 3.125 μM (Fig. 4e). Based on colorimetric determination of heavy metal ions in aqueous matrix, several paper-based biosensor systems have been successfully developed^38,39^. The deepening of purple recognized to the naked eye might enable the violacein-based biosensor a robust, paper-based analytical device for monitoring high concentrations of toxic Cd(II).

### Response of violacein-based biosensor toward bioavailable Cd(II) in environmental water

**Figure 5:**
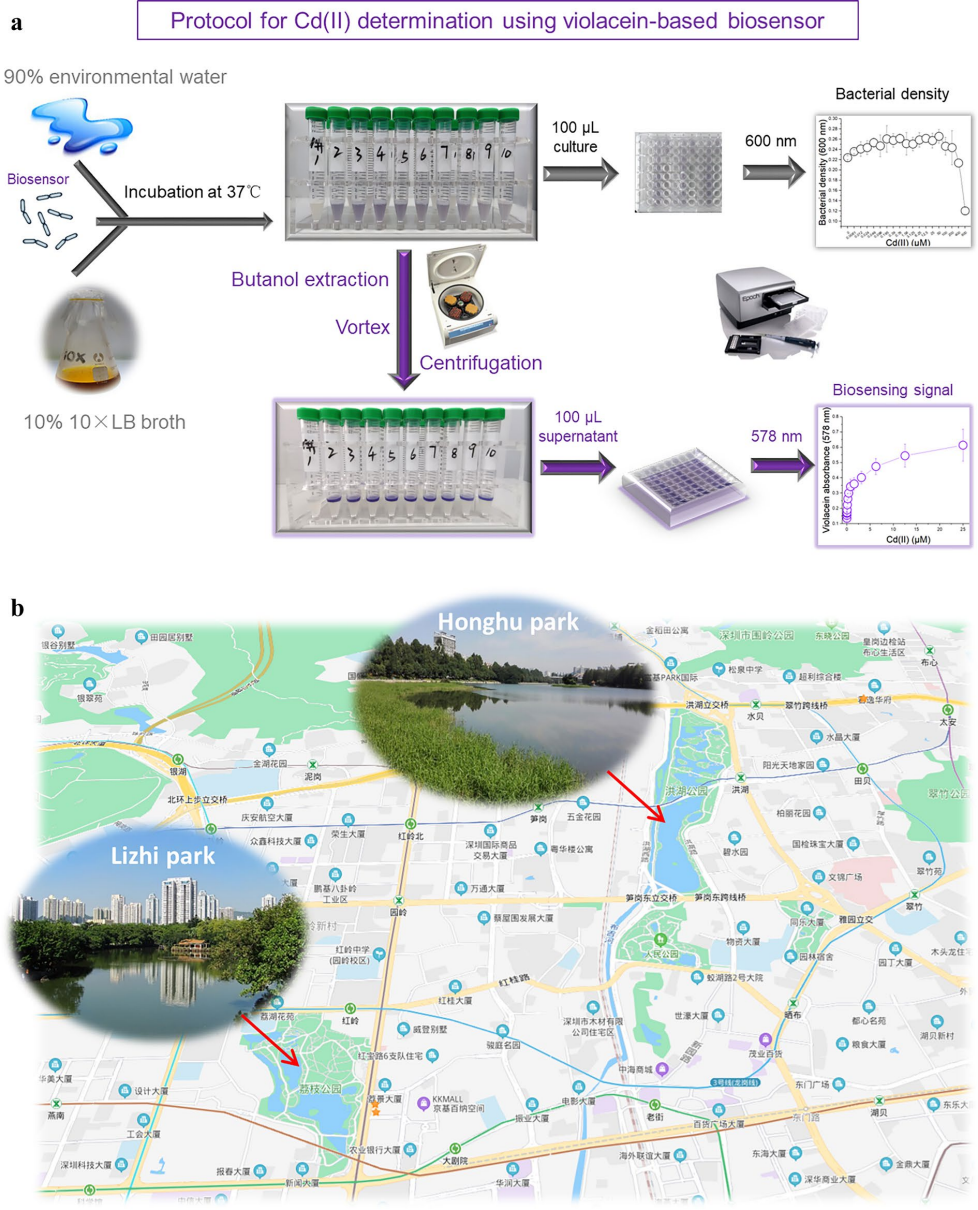

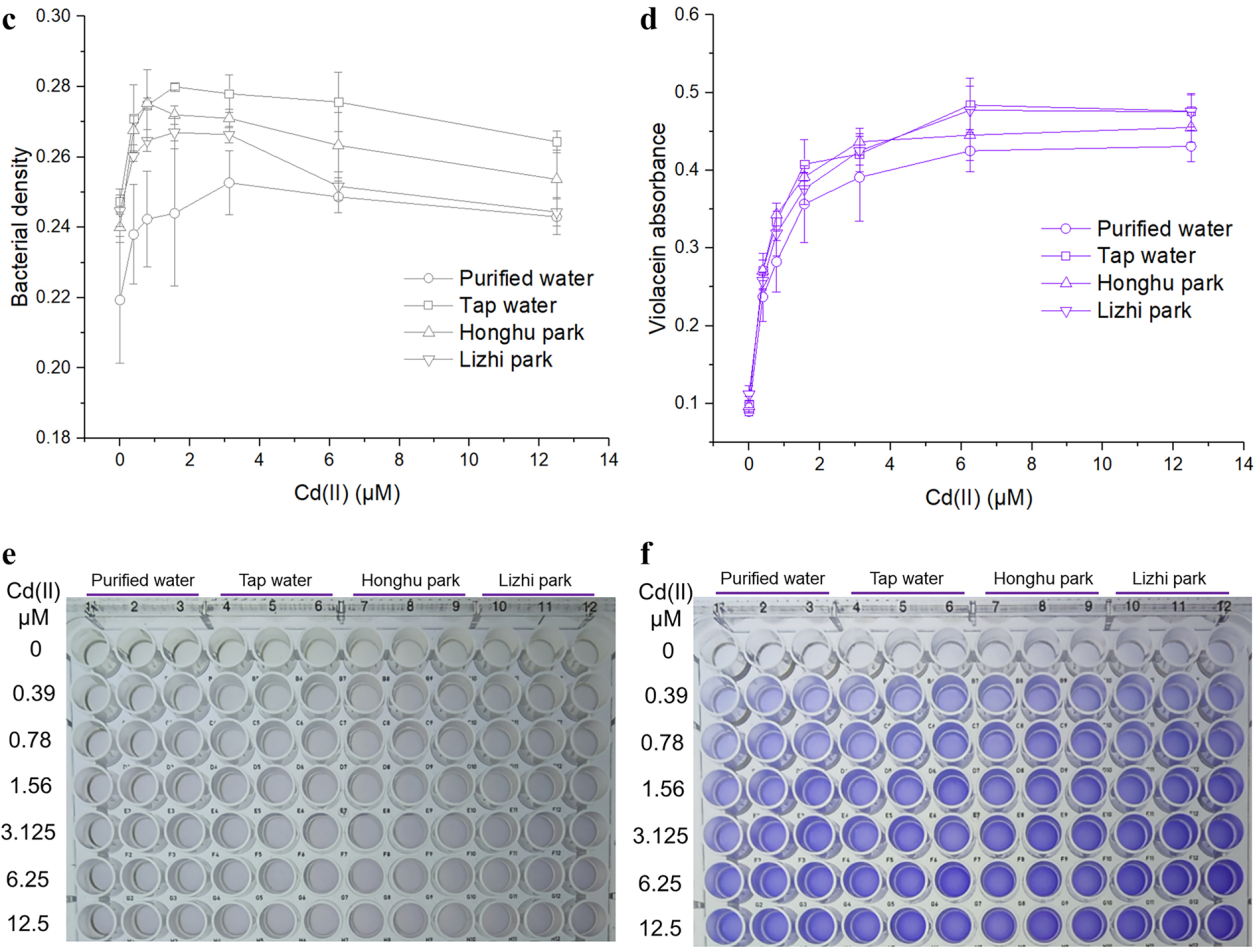
Determination of soluble Cd(II) in artificially polluted environmental water samples. (**a)** The detailed protocol for measurement of bioavailable Cd(II) using violacein-based biosensor. (**b)** Environmental water was sampled from two local parks in downtown Shenzhen (map picture from Baidu Maps online). Early exponential-phase TOP10/pPcad-vio cultured in LB medium prepared using four water samples was exposed to elevated concentrations of Cd(II). After being cultured at 37 °C for 1.5 h, the bacterial densities (**c**) and the violacein-derived signals (**d**) were measured at 600 nm and 578 nm, respectively. A representative picture of the cultures (**e**) and the butanol extraction phases (**f**) with three independent assays in 96-well plates, showing that the production of violacein was positively correlated with the concentrations of Cd(II) spiked in environmental water samples.

A procedure is proposed to simplify and cost-effectively detect bioavailable Cd(II) in environmental water samples (Fig. 5a). Minimal instruments are employed in the testing process, which is composed of preparation of culture medium, incubation, butanol extraction, and colorimetric determination after centrifugation. To compare the performance of biosensors responding to environmental water, different water samples including purified water, tap water, and two kinds of environmental surface water (Fig. 5b) were used to prepare culture broths. Total cadmium in these water samples was first demonstrated to be below the detection limit (0.004 μM) in atomic absorption spectrometry^40,41^. The early exponential-phase biosensor cells cultured in these mediums were exposed to different concentrations of Cd(II) at 37 °C for 1.5 h. The increase in turbidity measured as optical density at 600 nm was entirely attributed to intracellular pigment accumulation (Fig. 5c). The signals derived from violacein in the butanol phase significantly enhanced with the increase of concentration of spiked Cd(II). Importantly, the rising trends of pigment signals were all similar in four groups (Fig. 5d). The result showed that complex matrix in environmental water samples exerts slight influence on the biosynthesis of violacein triggered by bioavailable Cd(II). Owning to the hydrophobic characteristics of violacein, the color deepening in bacterial culture was not obvious (Fig. 5e). However, the color deepening in butanol phase became perceptible to the naked eye, and the results of three independent groups also showed excellent consistency (Fig. 5f).

## Conclusion

In the present work, a bacterial biosensor selectively responding to toxic cadmium using a visual pigment as the output signal was developed. Direct visual feedback combined with colorimetric test was used in quantitatively determining bioavailable Cd(II). Although the background of the biosensor was a little high, the detection sensitivity was significantly higher than previously developed biosensors using fluorescence proteins. A proposed procedure was successfully used to detect soluble Cd(II) in environmental water samples. Our finding demonstrates that Cd(II)-induced violacein biosynthesis enables a fast-responding, low-cost, minimal-equipment biosensor which can be used to predict the ecotoxicology of heavy metals in the environment.

## References

[1] Kumar A (2021). Bio-remediation approaches for alleviation of cadmium contamination in natural resources. Chemosphere.

[2] Hui CY, Guo Y, Liu L, Yi J (2021). Recent advances in bacterial biosensing and bioremediation of cadmium pollution: A mini-review. World J. Microbiol. Biotechnol..

[3] Gupta N, Renugopalakrishnan V, Liepmann D, Paulmurugan R, Malhotra BD (2019). Cell-based biosensors: Recent trends, challenges and future perspectives. Biosens. Bioelectron..

[4] Rittle J, Field MJ, Green MT, Tezcan FA (2019). An efficient, step-economical strategy for the design of functional metalloproteins. Nat. Chem..

[5] Guo Y (2021). Development of cadmium multiple-signal biosensing and bioadsorption systems based on artificial *cad* operons. Front. Bioeng. Biotechnol..

[6] Kang Y, Lee W, Jang G, Kim BG, Yoon Y (2018). Modulating the sensing properties of *Escherichia coli*-based bioreporters for cadmium and mercury. Appl. Microbiol. Biotechnol..

[7] Kumar S, Verma N, Singh A (2017). Development of cadmium specific recombinant biosensor and its application in milk samples. Sens. Actuators B Chem..

[8] Bereza-Malcolm L, Aracic S, Kannan R, Mann G, Franks AE (2017). Functional characterization of Gram-negative bacteria from different genera as multiplex cadmium biosensors. Biosens. Bioelectron..

[9] Kim HJ (2016). Development of a highly specific and sensitive cadmium and lead microbial biosensor using synthetic CadC-T7 genetic circuitry. Biosens. Bioelectron..

[10] Wang D (2020). Visual detection of Hg(2+) by manipulation of pyocyanin biosynthesis through the Hg(2+)-dependent transcriptional activator MerR in microbial cells. J. Biosci. Bioeng..

[11] Hui CY (2021). Indigoidine biosynthesis triggered by the heavy metal-responsive transcription regulator: A visual whole-cell biosensor. Appl. Microbiol. Biotechnol..

[12] Joe MH (2012). Pigment-based whole-cell biosensor system for cadmium detection using genetically engineered *Deinococcus radiodurans*. Bioprocess Biosyst. Eng..

[13] Hui CY (2022). A tailored indigoidine-based whole-cell biosensor for detecting toxic cadmium in environmental water samples. Environ. Technol. Innov..

[14] Cauz ACG (2019). Violacein targets the cytoplasmic membrane of bacteria. ACS Infect. Dis..

[15] Cress BF (2016). Rapid generation of CRISPR/dCas9-regulated, orthogonally repressible hybrid T7-lac promoters for modular, tuneable control of metabolic pathway fluxes in *Escherichia coli*. Nucleic Acids Res..

[16] Tong Y, Zhou J, Zhang L, Xu P (2021). A golden-gate based cloning toolkit to build violacein pathway libraries in *Yarrowia lipolytica*. ACS Synth. Biol..

[17] Yang D, Park SY, Lee SY (2021). Production of rainbow colorants by metabolically engineered *Escherichia coli*. Adv. Sci..

[18] McNerney MP, Michel CL, Kishore K, Standeven J, Styczynski MP (2019). Dynamic and tunable metabolite control for robust minimal-equipment assessment of serum zinc. Nat. Commun..

[19] Guo Y, Hui CY, Liu L, Chen MP, Huang HY (2021). Development of a bioavailable Hg(II) sensing system based on MerR-regulated visual pigment biosynthesis. Sci. Rep..

[20] Hui CY (2020). Genetic control of violacein biosynthesis to enable a pigment-based whole-cell lead biosensor. RSC Adv..

[21] Hui CY (2021). Detection of bioavailable cadmium by double-color fluorescence based on a dual-sensing bioreporter system. Front. Microbiol..

[22] Park H, Park S, Yang YH, Choi KY (2021). Microbial synthesis of violacein pigment and its potential applications. Crit. Rev. Biotechnol..

[23] Lee SW, Glickmann E, Cooksey DA (2001). Chromosomal locus for cadmium resistance in *Pseudomonas putida* consisting of a cadmium-transporting ATPase and a MerR family response regulator. Appl. Environ. Microbiol..

[24] Hui CY, Guo Y, Li H, Chen YT, Yi J (2022). Differential detection of bioavailable mercury and cadmium based on a robust dual-sensing bacterial biosensor. Front. Microbiol..

[25] Jung J, Lee SJ (2019). Biochemical and biodiversity insights into heavy metal ion-responsive transcription regulators for synthetic biological heavy metal sensors. J. Microbiol. Biotechnol..

[26] Waldron KJ, Rutherford JC, Ford D, Robinson NJ (2009). Metalloproteins and metal sensing. Nature.

[27] Tao HC, Peng ZW, Li PS, Yu TA, Su J (2013). Optimizing cadmium and mercury specificity of CadR-based *E. coli* biosensors by redesign of CadR. Biotechnol. Lett..

[28] Zhang NX (2021). Versatile artificial mer operons in *Escherichia coli* towards whole cell biosensing and adsorption of mercury. PLoS ONE.

[29] Wu CH, Le D, Mulchandani A, Chen W (2009). Optimization of a whole-cell cadmium sensor with a toggle gene circuit. Biotechnol. Prog..

[30] Jia X, Liu T, Ma Y, Wu K (2021). Construction of cadmium whole-cell biosensors and circuit amplification. Appl. Microbiol. Biotechnol..

[31] Riether KB, Dollard MA, Billard P (2001). Assessment of heavy metal bioavailability using *Escherichia coli* zntAp::lux and copAp::lux-based biosensors. Appl. Microbiol. Biotechnol..

[32] Ivask A (2004). Recombinant luminescent bacterial sensors for the measurement of bioavailability of cadmium and lead in soils polluted by metal smelters. Chemosphere.

[33] Biran I, Babai R, Levcov K, Rishpon J, Ron EZ (2000). Online and *in situ* monitoring of environmental pollutants: Electrochemical biosensing of cadmium. Environ. Microbiol..

[34] Gireesh-Babu P, Chaudhari A (2012). Development of a broad-spectrum fluorescent heavy metal bacterial biosensor. Mol. Biol. Rep..

[35] Yoon Y (2016). Use of tunable whole-cell bioreporters to assess bioavailable cadmium and remediation performance in soils. PLoS ONE.

[36] Wang B, Barahona M, Buck M (2013). A modular cell-based biosensor using engineered genetic logic circuits to detect and integrate multiple environmental signals. Biosens. Bioelectron..

[37] Yoon Y (2016). Simultaneous detection of bioavailable arsenic and cadmium in contaminated soils using dual-sensing bioreporters. Appl. Microbiol. Biotechnol..

[38] Grawe A (2019). A paper-based, cell-free biosensor system for the detection of heavy metals and date rape drugs. PLoS ONE.

[39] Allafchian AR, Farajmand B, Koupaei AJ (2018). A paper-based analytical device based on combination of thin film microextraction and reflection scanometry for sensitive colorimetric determination of Ni(II) in aqueous matrix. Bull. Environ. Contam. Toxicol..

[40] Hui C (2018). Surface display of PbrR on *Escherichia coli* and evaluation of the bioavailability of lead associated with engineered cells in mice. Sci. Rep..

[41] Hui CY, Guo Y, Yang XQ, Zhang W, Huang XQ (2018). Surface display of metal binding domain derived from PbrR on *Escherichia coli* specifically increases lead(II) adsorption. Biotech. Lett..

